# Identification of landscape features influencing gene flow: How useful are habitat selection models?

**DOI:** 10.1111/eva.12389

**Published:** 2016-06-03

**Authors:** Gretchen H. Roffler, Michael K. Schwartz, Kristy L. Pilgrim, Sandra L. Talbot, George K. Sage, Layne G. Adams, Gordon Luikart

**Affiliations:** ^1^ US Geological SurveyAlaska Science CenterAnchorageAKUSA; ^2^ Wildlife Biology ProgramDepartment of Ecosystem Sciences and ConservationCollege of Forestry and ConservationUniversity of MontanaMissoulaMTUSA; ^3^ US Forest Service Rocky Mountain Research StationMissoulaMTUSA; ^4^ Flathead Lake Biological StationUniversity of MontanaPolsonMTUSA; ^5^ Fish and Wildlife Genomics GroupDivision of Biological SciencesUniversity of MontanaMissoulaMTUSA; ^6^ Present address: Alaska Department of Fish and GameDivision of Wildlife ConservationDouglasAKUSA

**Keywords:** dispersal, landscape genetics, multiple regression on distance matrices, *Ovis dalli dalli*, population connectivity, resistance surfaces, resource selection function

## Abstract

Understanding how dispersal patterns are influenced by landscape heterogeneity is critical for modeling species connectivity. Resource selection function (RSF) models are increasingly used in landscape genetics approaches. However, because the ecological factors that drive habitat selection may be different from those influencing dispersal and gene flow, it is important to consider explicit assumptions and spatial scales of measurement. We calculated pairwise genetic distance among 301 Dall's sheep (*Ovis dalli dalli*) in southcentral Alaska using an intensive noninvasive sampling effort and 15 microsatellite loci. We used multiple regression of distance matrices to assess the correlation of pairwise genetic distance and landscape resistance derived from an RSF, and combinations of landscape features hypothesized to influence dispersal. Dall's sheep gene flow was positively correlated with steep slopes, moderate peak normalized difference vegetation indices (NDVI), and open land cover. Whereas RSF covariates were significant in predicting genetic distance, the RSF model itself was not significantly correlated with Dall's sheep gene flow, suggesting that certain habitat features important during summer (rugged terrain, mid‐range elevation) were not influential to effective dispersal. This work underscores that consideration of both habitat selection and landscape genetics models may be useful in developing management strategies to both meet the immediate survival of a species and allow for long‐term genetic connectivity.

## Introduction

Landscape heterogeneity influences the distribution of animals through the spatial configuration and degree of connectivity of preferred habitats. Dispersal and seasonal movement patterns among geographic areas could be impeded by physical barriers or by large expanses of habitats a species avoids, or conversely facilitated by continuity of preferred habitats (McRae and Beier 2007; Rudnick et al. 2012). In turn, gene flow across landscapes resulting from dispersal influences patterns of population genetic structuring (Manel et al. 2003; Spear et al. 2010; Wagner and Fortin 2012). To understand how landscape heterogeneity influences effective dispersal and gene flow, models that characterize habitat use are increasingly employed to inform landscape genetics analyses. Habitat‐based and landscape genetics approaches are different but complimentary, and combined can identify important habitats for different life history requirements of a species. Integrated habitat and landscape genetics models also provide valuable information for resource managers to promote connectivity between critical habitats through designing corridors and conservation areas (Chetkiewicz and Boyce 2009).

A major objective of the field of landscape genetics is to assess the effects of landscape features on genetic connectivity (Manel et al. 2003). A landscape genetics approach may be used to quantify the effects of landscape features on spatial patterns of neutral genes (Holderegger and Wagner 2008) and infer breeding and dispersal movements (Storfer et al. 2007). Landscape genetic models can measure how landscape features influence gene flow by quantifying the resistance of environmental characteristics on dispersal (Spear et al. 2010). A range of values are assigned to habitat characteristics using resistance surfaces; low resistance values facilitate animal movement (with little restraint), while high resistance values restrict animal movement and thus gene flow. Hypothesized resistance values are often based on expert opinion (Coulon et al. 2004; Epps et al. 2007; Shirk et al. 2010) or empirical data (e.g., survey or telemetry locations; Schwartz et al. 2009; Spear et al. 2010; Shafer et al. 2012) and are then tested with genetic data.

Resource selection functions provide information on the relative probability of use of a given set of resources by an organism (Manly et al. 2002). Instead of assessing single landscape variables independently, RSFs can be used as a resistance layer to evaluate the combined effects of landscape characteristics on genetic structure. Application of the inverse values of RSFs to parameterize resistance surfaces assumes that higher quality (and more frequently used) habitat presents lower costs (or ‘friction’) for movement. Habitat suitability or RSF models have been used to parameterize resistance surfaces to assess the influence of habitat features on least‐cost path corridors (Chetkiewicz and Boyce 2009; Pullinger and Johnson 2010), patterns of gene flow using graph theory modeling (Garroway et al. 2008), and landscape genetics models (McRae and Beier 2007; Spear et al. 2010; Epps et al. 2013). The combination of methods provides a powerful analytical platform, but is based on the assumption that the ecological factors that drive habitat selection are similar to those that influence dispersal and gene flow.

Indeed, habitat selection and landscape genetics models often measure different ecological processes at different temporal and spatial scales (Table 1). It is important to recognize that suitable habitat within a home range may not equate to low resistance for dispersal and gene flow (Braunisch et al. 2010; Spear et al. 2010). For example, habitat features required to maintain nutritional condition and safety from predators within an organism's home range could vary substantially from those used for movement to seasonal breeding ranges or for exploratory movements. Habitat selection and landscape genetic models also reflect different timescales. Genetic data integrates multiple generations of breeding dispersal and thus represents the average long‐term effective dispersal across a landscape. On the other hand, RSF models generally account for daily or seasonal movement patterns and use current resource data to characterize habitats within a contemporary time frame.

**Table 1:** Comparison of resource selection function (RSF) and landscape genetics models in terms of the ecological processes they measure at different spatial and temporal scales, and model assumptions

	RSF	Landscape genetics
Ecology	Food, safety	Effective dispersal
	Measures use and relative importance of habitat variables	Measures how habitat variables influence genetic connectivity
Time	Shorter time frame	Longer time frame—multiple generations
	Seasonal	Gene flow in populations with small effective population size (Ne) reflects more recent dispersal than populations with large Ne
Space	Structural connectivity	Functional connectivity of individuals and populations across landscapes
	Broad‐scale: Seasonal home ranges within the species or population home ranges	
	Fine‐scale: Habitat selection within seasonal home ranges	
Assumptions	RSF is proportional to the probability of use	Pairwise genetic distance is informative of gene flow
	Resource units are sampled randomly and independently	Genetic differentiation results from habitat heterogeneity, instead of historic demographic events (e.g., population bottlenecks)
	Resources are constant throughout time period of study	
	Availability of resources does not vary	Gene flow reflects movement of individuals that successfully reproduce (or their gametes)
	Organisms select resources according to how they will benefit from them	If sex‐biased dispersal is not directly accounted for, the assumption is that patterns of gene flow are similar for males and females
	Probability of selection is related to habitat quality	

One of the benefits of RSF models is that they may be created using data from multiple spatial scales (Johnson 1980; Hebblewhite et al. 2008), but generally focus on selection of specific resources within a seasonal or home range. Landscape genetics models may also be analyzed using data collected at multiple spatial scales (e.g., Wasserman et al. 2010 but commonly estimate interindividual genetic distance, or genetic distance among networks of populations to assess population‐wide genetic connectivity. Thus, landscape genetics models provide information on dispersal and gene flow across space (functional connectivity; Manel et al. 2003; Holderegger and Wagner 2008), whereas RSFs measure the use and relative importance of habitat variables (Manly et al. 2002) and provide information on configuration of habitats (structural connectivity; Tischendorf and Fahrig 2000; Brooks 2003; Holderegger and Wagner 2008).

Recent research demonstrates a range of correlation between gene flow and the landscape features identified in RSFs or other habitat models, from positive (Epps et al. 2007; Wang et al. 2008; Shafer et al. 2012; Weckworth et al. 2013), to little or no correlation (Braunisch et al. 2010; Wasserman et al. 2010; Reding et al. 2013). Here, we integrated RSF and landscape resistance modeling approaches to determine whether the relative probability of habitat selection predicts gene flow in Dall's sheep (*Ovis dalli dalli*). Dall's sheep are habitat specialists, which like many alpine species in northern latitudes may be vulnerable to climate change, thus characterizing seasonal habitats and the degree of connectivity among them is essential for long‐term management.

Our RSF models included landscape variables hypothesized to be of ecological importance to sheep fitness and distribution and were developed using survey data over a broad, heterogeneous landscape in southcentral Alaska (Appendix S1). We used empirical data from the top RSF model (the inverse value of the relative probability of habitat selection for each landscape pixel) to parameterize resistance surfaces representing our hypothesized relationships between gene flow and landscape features. Using the same suite of habitat covariates that were used to evaluate habitat selection in Dall's sheep, we constructed competing resistance models of genetic distance and compared these models to the RSF model. Few studies employ this two‐stage approach wherein competing resistance models are constructed using empirical data and the top model is identified using model selection procedures and genetic distance data (Zeller et al. 2012). We predicted that the same characteristics that influence Dall's sheep seasonal habitat selection also influence patterns of longer‐term gene flow. We further predicted that the top models would be more correlated with genetic distance than either geographic distance alone, or major landscape barriers which could impede dispersal.

## Methods

### Study area

**Figure 1:**
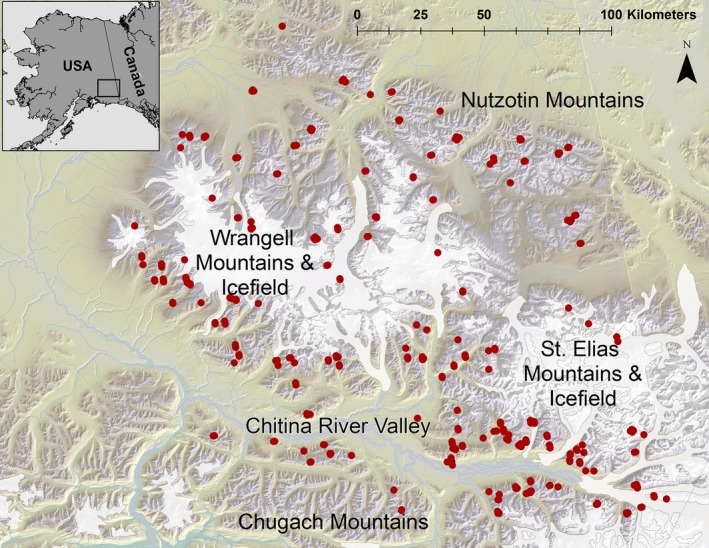
Collection locations of Dall's sheep genetic samples (*n *=* *301) in Wrangell‐St. Elias National Park and Preserve, Alaska, 2007–2009.

Wrangell‐St. Elias National Park and Preserve (WRST) in southcentral Alaska is an important region for Dall's sheep, encompassing approximately 28 000 km^2^ of contiguous sheep habitat. There are an estimated 11 000–14 500 Dall's sheep in WRST (Schmidt and Rattenbury 2013), equivalent to 15% of the subspecies' population. The study area extends geographically from maritime to interior habitats across multiple mountain ranges (Fig. 1) and therefore contains gradients of temperature (mean annual temperature = −10–18°C) and precipitation (mean annual precipitation = 300–3000 mm). The Chugach Range in the southern portion of the study area is mesic, the St. Elias and Wrangell Ranges in the central portion of WRST represent a transitional climate zone, and the Nutzotin Range is xeric. These mountain ranges are separated by potential barriers to sheep dispersal such as large ice fields (e.g., ≤120 km long, >40 km wide), glaciers (≤40 km long, 1–10 km wide), major river valleys, and low‐elevation (<1000 m) forested areas (Fig. 1).

### Genetic sample collections, genotyping, and genetic distance

Sample collections are described in Roffler et al. (2014). Briefly, we collected fresh feces in late summer 2007–2009 from randomly selected 50‐km² grid cells throughout the study area to distribute samples across the landscape. We also collected muscle samples and harvest locations from mature rams (≥3/4 curl) taken during hunting season (10 August–20 September), 2007–2009. Using extracted DNA, we genotyped 301 sheep at 15 microsatellite loci, performed standard tests of genetic variation and diversity, and assessed spatial patterns of genetic structure (Roffler et al. 2014). We tested for departures from selective neutrality using an F_ST_ outlier approach (Beaumont and Nichols 1996) and conservatively removed loci if they were consistently flagged as outliers in pairwise population comparisons. Applying these genetic markers to this landscape genetics study, we calculated the pairwise genetic distance between individuals bootstrapped 2000 times with MSA v4.05 (Dieringer and Schlötterer 2003) according to the proportion of shared alleles (*D*_ps_; Bowcock et al. 1994).

### Resource selection function models

**Figure 2:**
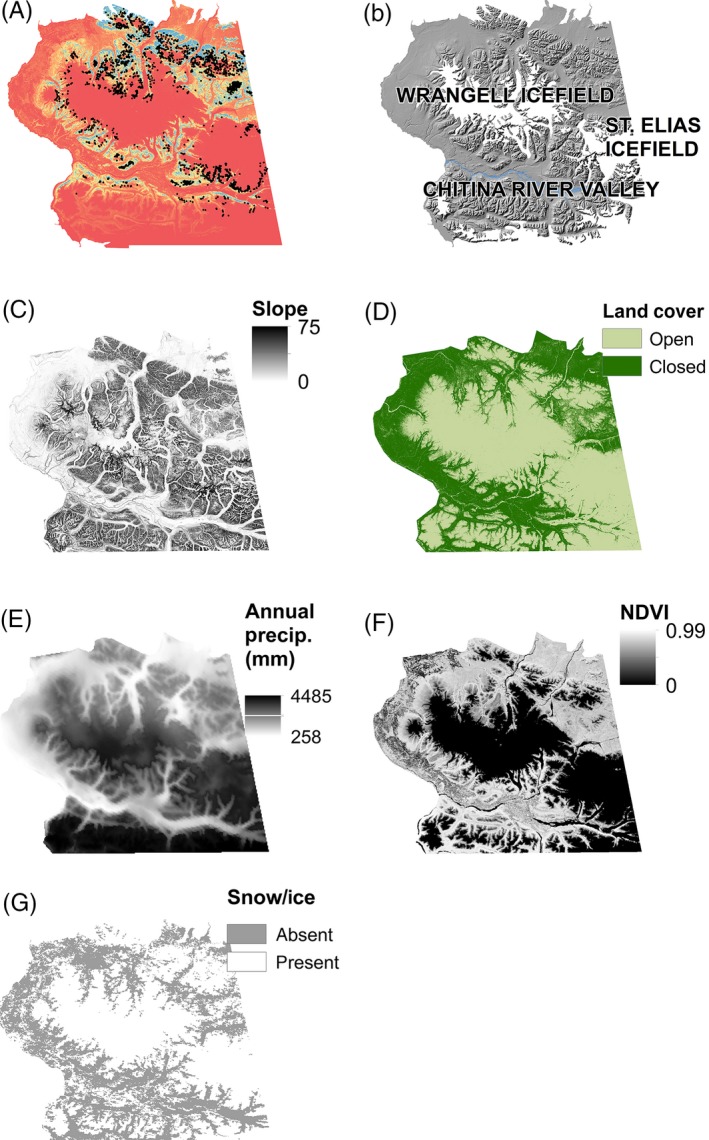
(A) Dall's sheep locations 1983–2011 (*n *=* *2587) used to build the summer resource selection functions, and the predicted values for probability of habitat use, (B) low‐elevation valleys and ice formations tested as potential barriers to gene flow, (C) slope, (D) open‐ and closed‐canopy habitat categories, (E) mean annual precipitation (mm), (F) mean peak annual NDVI value 2001–2011, (G) presence of snow on October 1, Wrangell‐St. Elias National Park and Preserve, Alaska.

We used empirical data to inform the construction of resistance surfaces of gene flow by modeling Dall's sheep habitat resource selection (Appendix S1). In brief, we estimated summer habitat selection of WRST sheep with RSFs (Boyce et al. 2002; Manly et al. 2002) at the second‐order scale (landscape; Johnson 1980), and our design corresponded to surveys with population level information about use and availability within seasonal home ranges (Appendix S1). Models included biotic and abiotic habitat covariates (topographic, climatic, land cover, normalized difference vegetation index [NDVI]) that were previously reported to be influential to Dall's sheep distribution (Table S1). We compared habitat at used and available sheep group locations from summer aerial surveys during 1983 – 2011 with generalized logistic regression (Hosmer and Lemeshow 2000; Appendix S1). We estimated the relative probability of use with the exponential approximation of the logistic regression model (Manly et al. 2002; Lele and Keim 2006; Appendix S1). To project landscape resistance spatially, we first scaled the predicted values from the relative probability of use model (eq. 1) between 0 and 1 (Johnson et al. 2004). We then calculated the inverse value of the scaled predicted probabilities using ArcGIS Spatial Analyst Raster Calculator in ArcMap 10.1 (ESRI, Redlands, CA, USA) at the resolution of 120‐m^2^ pixel (Fig. 2).

### Landscape resistance surfaces

We used an individual‐based, spatially explicit landscape genetics approach to determine the best predictor for genetic connectivity of individual Dall's sheep. We developed resistance surfaces derived from the inverse of the summer habitat selection RSF described above, of each individual habitat covariate in the RSF model, and for additional landscape variables hypothesized to affect Dall's sheep gene flow (see below and Table S1). We estimated pairwise resistance distances between all pairs of individuals for each landscape variable using CIRCUITSCAPE v4.0 (McRae and Beier 2007), which applies principles of circuit theory to assess connectivity across landscapes with hypothesized levels of resistance or conductivity. Circuit theory accommodates simultaneous modeling and ranking of all possible pathways of connectivity, accounting for varying levels of resistance due to path size. We conducted CIRCUITSCAPE analyses in a pairwise mode, with individual locations set as focal nodes, and connections were allowed between all eight surrounding cells of each pixel. We represented pairwise geographic distance between all individual sheep with a distance matrix (all cells in the resistance surface = 1), which served as the null model (referred to henceforth as Euclidean distance) against which the more complex landscape surfaces incorporating landscape heterogeneity were compared.

In addition to the habitat covariates included in the RSF model, we hypothesized that October 1 snow cover, a variable representing conditions affecting movements of sheep at the onset of winter and prior to the rut, could influence genetic connectivity (Table S1). Snow cover on October 1 was derived from daily gridded (500‐m resolution) Terra MODIS snow cover imagery (2001–2012) and algorithms to determine the spatial extent of persistent snow (Zhu and Lindsay 2013). We also represented geographic features within the WRST landscape which have been shown to present barriers to Dall's sheep gene flow, primarily the Wrangell and St. Elias ice fields and glaciers, and the lower Chitina River valley (Fig. 1), which aligned geographically with Bayesian clusters of individual Dall's sheep genotypes (Roffler et al. 2014). Resistance layers were produced using ArcGIS 10.1 by clipping each landscape covariate layer to the study area. Using the Spatial Analyst Raster Calculator, each 120‐m^2^ pixel in each landscape covariate layer was coded with a value of 1 if hypothesized to be low resistance and >1 (e.g., 2–500, see below) if high resistance. Several habitat covariates (peak NDVI, elevation, ruggedness, slope, and precipitation) had a nonlinear relationship with Dall's sheep habitat selection (Appendix S1), and thus, we expected they would also have nonlinear resistance relationship. We accommodated the landscape resistance curves with mathematical functions reflecting RSF variable shapes and coefficient values and reclassified the variable raster data into resistance surfaces (Appendix S2).

Assigning resistance values to landscape features is challenging as the effects of different surfaces on the modeled organisms' dispersal, survival, and reproductive capacities are generally unknown (Spear et al. 2010). To contend with this issue, we used an optimization framework (Tucker 2013) to select the optimal resistance for each landscape variable independently over a wide range of values (2–500) using partial Mantel tests to determine whether genetic and landscape resistance matrix relationships remained significant while controlling for the effects of Euclidean distance (Smouse et al. 1986). Thus, each landscape covariate resistance layer was scaled between 1–2 and 1–500, to represent this potential range of hypothetical resistance values. To identify the resistance value for each variable which maximized the relationship with genetic distance (i.e., the optimum resistance value), we ascertained the asymptote of the curve (rate of change <5%) of the partial Mantel *r* (*r*_pm_) plotted against the range of resistance values. While the use of partial Mantel tests has been debated (Balkenhol et al. 2009; Legendre and Fortin 2010), specifically the reliance on potentially biased significance tests (Graves et al. 2013), partial Mantel tests are an appropriate tool for characterizing the data distribution and shape of the landscape genetics relationships (Legendre and Fortin 2010) and still offer valuable initial tests especially when complemented with other analyses (Cushman et al. 2013). Further, we did not rely on partial Mantel significance tests in the optimization procedure and considered significance test in tandem with other analyses results for the landscape resistance model evaluation. The importance of univariate and multivariate optimization has been demonstrated (Shirk et al. 2010); therefore, we used the optimized univariate resistance surfaces in the multivariate candidate model evaluation process and to select a single optimal multivariate resistance surface (Tucker 2013). Univariate and partial Mantel tests were conducted using the *Ecodist* package (Goslee and Urban 2007) in R (version 2.13.0; R Development Core Team 2011).

### Model selection and evaluation

We constructed *a priori* models using combinations of the landscape resistance surfaces, and information theoretic criteria for model selection. We removed Euclidean distance from resistance distances for each variable prior to all model selection procedures because Euclidean distance is factored into effective resistance in CIRCUITSCAPE. We excluded significantly correlated landscape variables indicated by a Pearson's correlation of *r *≥* *0.7 (Hosmer and Lemeshow 2000), or with variance inflation factors (VIF) >10 in the global model and >5 in the final candidate models (McCullough and Nelder 1989). We standardized optimum resistances using a z‐transformation so that parameter estimates for each variable would be comparable.

We used multiple regression of distance matrices (MRDM; Legendre et al. 1994) on the optimized distance matrices to determine the relative importance of landscape resistance distances (explanatory variables) and genetic distance (the dependent variable) between individuals. This method has been demonstrated to retain a good balance between type 1 error and power and has high levels of accuracy compared with other methods (Balkenhol et al. 2009). We performed tests using 9999 permutations and 1000 iterations to obtain bootstrap confidence intervals. We developed multiple candidate models to determine the landscape resistance model best supported by the genetic data. To evaluate relative model performance, we also used linear regression and Akaike's information criterion (AICc) corrected for small sample sizes and the associated AICc weights (*w*_*i*_) to select the top model (Anderson and Burnham 2002). Although use of information theory has been criticized due to potential nonindependence of pairwise data, it has been used as a means to rank multivariate models explaining genetic distance because the error is assumed to be equal for each model, thus not affecting ranking ability (Garroway et al. 2008; Richardson 2012; Engler et al. 2014). However, as this error may possibility affect confidence of assessing model significance, we conservatively evaluated results of multiple assessment methods in addition to AICc weights for final model selection (described below). We excluded models in the top set that contained uninformative parameters (differed from the top model by 1 parameter and did not improve the AICc score by at least 2; Arnold 2010). We used 85% confidence intervals to eliminate variables that overlapped zero, indicating that they were also uninformative to the model (Arnold 2010).

We converted the top model into a composite multivariate resistance surface using the resulting equation and model parameter values in the ArcGIS Spatial Analyst Raster Calculator. We obtained the parameter estimates using the untransformed variables to fit the top model. The parameter coefficient estimate was then multiplied by the resistance surface for each landscape variable, and each separate surface summed using Raster Calculator. This composite multivariate surface was then used to determine pairwise individual resistance distances with CIRCUITSCAPE. We then determined the correlation between genetic distance and resistance distance of the following models: (1) the top MRDM model, (2) the RSF model, and (3) the isolation‐by‐distance (IBD) model (i.e., genetic distance ~Euclidean distance). The strength of correlation was assessed with *r* and *r*_pm_ and model fit assessed with *R* ^2^. We expected the multivariate resistance surface would have a higher *r*_pm_ than the univariate surfaces if the modeling approach was effective.

To accept a model as explanatory of genetic distance, it was necessary that the partial Mantel test (the relationship between two matrices while controlling for the effects of a third matrix) have a significant result, and simultaneously the opposite test not be significant. For example, the correlation of genetic distance and resistance distance while controlling for Euclidean distance must be significant, while the correlation of genetic distance and Euclidean distance while controlling for resistance distance must not be significant (Cushman and Landguth 2010; Wasserman et al. 2010; Koen et al. 2011). This result would indicate that landscape resistance is driving genetic distance rather than IBD. This causal modeling has been demonstrated to differentiate between hypothetical models (i.e., landscape resistance and IBD) that are influential to the genetic distance as opposed to merely correlated (Cushman and Landguth 2010). We compared results of multiple methods (*r*_pm_, AICc, and *R* ^2^ of MRDM model) to compensate for limitations of either method used alone. All model selection statistics were performed in R.

Some limitations of Mantel tests include nonindependence of the distance data structure and inability to estimate the proportion of data variation explained by spatial structures such as landscape variables (Legendre and Fortin 2010). In order to bolster our analyses, we used distance‐based Moran's eigenvector maps (MEM; also referred to as PCNM; Borcard and Legendre 2002) and multivariate regression of genetic distance matrices and the landscape resistance surfaces, implemented in the MEMGENE R package (Galpern et al. 2014; Appendix S3). This approach differs from Mantel distance regressions as the explanatory variables are not transformed into distances. The creation of the MEM orthogonal spatial variables (with zero correlations) is accomplished through principal coordinate analysis of a truncated geographic distance matrix among sampling sites (Dray et al. 2006). The MEM eigenvectors are then used as explanatory variables and the genetic distance matrix as the dependent variable to identify the spatial component of genetic variation in the data. The amount of genetic variation explained by spatial pattern of each model was estimated as the adjusted coefficient of determination *R* ^2^ (adj *R* ^2^).

## Results

### Genotyping and genetic distance

We obtained complete 15‐locus genotypes of 301 sheep. There was no pattern across loci or among sampling locations of deviations from Hardy–Weinberg proportions (10 of 105 tests, after Bonferroni correction), and no significant linkage disequilibrium (*P* > 0.01). No loci fell outside the 99% confidence intervals when plotting total heterozygosity against F_ST_ (Roffler et al. 2014); thus, none were considered to be outliers and all loci were included in analyses. The number of alleles per locus ranged from 3.23 to 5.85 (mean = 4.66, SE = 0.373). The Mantel test demonstrated a significant positive correlation between geographic versus genetic distance (*r *=* *0.28, *P *=* *0.001), indicating a pattern of isolation by distance in WRST Dall's sheep.

### Resource selection function models

The top summer RSF model (Table S2) indicated that Dall's sheep select rugged, steep, and mid‐elevation terrain (between low‐elevation forest and tall shrub types and high‐level barren and persistent snow/ice types), intermediate levels of peak NDVI, and open habitat types (Appendix S1). Internal validation of the RSF model assessed through k‐fold cross‐validations demonstrated high Spearman's rank correlation coefficients averaged from five partitions (*r*_*s*_ = 0.997, *P *<* *0.001), suggesting the RSF is useful in predicting the relative probability of resource selection by Dall's sheep in WRST (Appendix S1).

### Landscape resistance models

All landscape variables reached an asymptote of optimal resistance values within the range of values tested and varied from 5 to 100 (Table S1). After partialling out the Euclidean distance in the univariate models, summer precipitation, October 1 snow, peak NDVI and slope had the strongest positive correlations with genetic distance (Table 2). Additional suspected barriers to Dall's sheep gene flow were not correlated with genetic distance (ice fields and glaciers, *r*_pm_ = −0.01; the lower Chitina River valley, *r*_pm_ = −0.11).

**Table 2:** Partial Mantel test results of the top univariate models comparing matrices of individual Dall's sheep genetic distance (Dps; Bowcock et al. 1994) and resistance distances (calculated using circuit theory), controlling for the effect of Euclidean distance, Wrangell‐St. Elias National Park and Preserve, Alaska

Landscape variable	Optimum resistance	Partial Mantel *r*_pm_	*P*
October 1 snow	50	0.13	0.001
Peak NDVI	50	0.09	0.001
Summer precipitation	50	0.13	0.001
Slope	50	0.05	0.004

The best multivariate model included elevation and open land cover in addition to 2 of the 4 univariate habitat variables that explained most of the genetic distance (peak NDVI and slope; Table 3). Dall's sheep gene flow was positively correlated with steep slopes, moderate values of peak NDVI, and open‐canopy habitat types and negatively correlated with mid‐elevations and heavy annual precipitation. The top model garnered all the support (*w*_*i* _= 1.00) once the candidate model list was refined to exclude uninformative and collinear parameters (Table 4). None of the 85% confidence intervals of the coefficients overlapped zero, and each parameter estimate was significantly different than expected by chance alone (Table 3).

**Table 3:** Parameter estimates (*β*), standard error (SE), permuted *P* values (*P*; the proportion of randomized parameter estimates greater than those based upon the original data), 85% confidence intervals (85% CI), for top ranked model explaining Dall's sheep gene flow, Wrangell‐St. Elias National Park and Preserve, Alaska

	*β*	SE	*P*	85% CI
Intercept	0.000	0.004	1.000	−0.006, 0.006
Elevation	−0.149	0.008	<0.001	−0.161, −0.137
Open habitat types	0.125	0.008	<0.001	0.113, 0.136
Peak NDVI	0.280	0.011	<0.001	0.265, 0.295
Annual mean precipitation	−0.232	0.006	<0.001	−0.241, −0.223
Slope	0.208	0.011	<0.001	0.193, 0.223

**Table 4:** (a) Ranked models from the top 12 candidate list explaining landscape resistance to Dall's sheep genetic distance (Dps; Bowcock et al. 1994), Wrangell‐St. Elias National Park and Preserve, Alaska. Partial Mantel coefficients (*r*_pm_) and *P* values are shown for genetic distance and the top multiple regression of distance matrices (MRDM) models controlling for the effects of Euclidean distance (ED), and Mantel (*r*) coefficients, and *P* values are shown for the isolation‐by‐distance (IBD) model. ΔAICc and Akaike weights (*w*_*i*_) for linear models and MRDM model fit (*R* ^2^) are in ranked order. (b) Mantel (*r*) coefficients and *P* values are shown for genetic distance and (1) the composite ecological resistance from the top MRDM model, and (2) the resource selection function (RSF) model. Partial Mantel coefficients (*r*_pm_) and *P* values for genetic distance and resistance distance are also shown controlling for the effects of the variable after the vertical bar

	*r*	*P*	*r*_pm_	*P*	ΔAICc	*w*_*i*_	*R* ^2^
(a) Model^a^							
elevation + open + p.NDVI + a.precip + slope			0.23	0.001	0	1	0.131
open + p.NDVI + s.precip + a.precip + slope + Oct.snow			0.01	0.384	110.28	0	0.129
p.NDVI + s.precip + a.precip + Oct.snow			0.01	0.394	197.68	0	0.128
elevation + open + rugged + slope + Oct.snow			0.01	0.460	1096.63	0	0.103
elevation + open + rugged + slope + Oct.snow + Chit + L.Chit + glacier			0.01	0.408	1458.93	0	0.11
elevation + rugged + slope + Chit + L.Chit + glacier			−0.07	1.000	1664.23	0	0.099
p.NDVI + s.precip + slope + Oct.snow			0.12	0.001	1718.62	0	0.098
s.precip			0.13	0.001	2517.47	0	0.082
IBD	0.28	0.001			2745.24	0	0.08
p.NDVI			0.09	0.001	2786.52	0	0.076
slope			0.05	0.004	2803.13	0	0.076
RSF			−0.06	1.000	3138.81	0	0.069
(b) Models							
GD~top MRDM	0.31	0.001					
GD~top MRDM│ED			0.23	0.001			
GD~ED│top MRDM			−0.17	1			
GD~RSF	0.26	0.001					
GD~RSF│ED			−0.06	0.966			
GD~ED│RSF			0.11	0.002			

a Variables described in Table S1 (open = open land cover class; p. NDVI = peak normalized difference vegetation index; a. precip = mean annual precipitation; s. precip = mean summer precipitation; Chit. = Chitina River Valley; L. Chit. = Lower Chitina River Valley).

**Figure 3:**
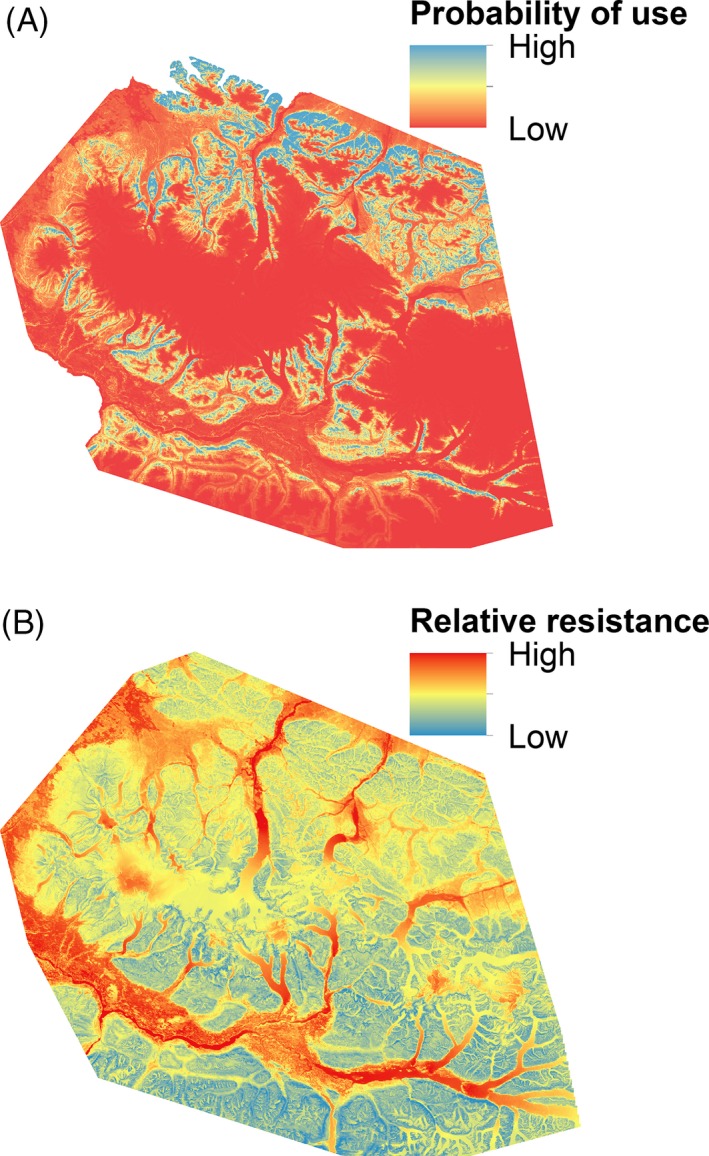
The inverse of predicted values for Dall's sheep summer resource selection functions (A), and the best resistance surface based on genetic data (B), Wrangell‐St. Elias National Park and Preserve, Alaska.

Using the composite resistance surface derived from the top model parameter estimates, we depicted an overall model of resistance to Dall's gene flow (Fig. 3). In addition to ranking the highest in AICc model selection procedures (Table 4a), this resistance model had the best model fit (*R* ^2 ^= 0.131) in comparison with both the IBD and RSF model (*R* ^2 ^= 0.077 and 0.069, respectively), although all MRDR models had fairly low *R* ^2^ values (Table 4a). As further support, the partial Mantel test controlling for the effects of Euclidean distance was significant (*r*_pm_ = 0.23, *P *=* *0.001), while the reverse test (the effect of Euclidean distance on genetic distance controlling for the top resistance model) was not (*r*_pm_ = −0.17, *P *=* *1.000; Table 4b). The IBD model performed poorly in the AICc model selection procedure (Table 4a) and was less correlated to genetic distance than the composite top MRDM model resistance (*r *=* *0.28 and 0.31, respectively). These results suggest that landscape resistance is a more important predictor of genetic distance than IBD.

We identified 22 positive MEM variables all of which occurred on the largest end of the eigenvalue spectrum, indicating that genetic variation is structured at the broadest spatial scales in our study area. We then compared the proportion of spatial genetic variation explained by each of the landscape resistance surfaces and the Euclidean distance model. The MEM eigenvectors derived from spatial patterns in the slope, annual precipitation, and peak NDVI resistance surfaces explained the highest proportion of spatial genetic variation (adj *R* ^2 ^= 0.195, *P *<* *0.001; adj *R* ^2 ^= 0.194, *P *<* *0.001; adj *R* ^2 ^= 0.187, *P *<* *0.001, respectively). RSF was also highly ranked in the MEM models (adj *R* ^2 ^= 0.191, *P *<* *0.001). Open‐canopy habitat types, October 1 snow, and elevation explained relatively less variation, but were still significant (adj *R* ^2 ^= 0.144, *P *<* *0.001; adj *R* ^2 ^= 0.137, *P *<* *0.001; adj *R* ^2 ^= 0.122, *P *<* *0.001, respectively). The Euclidean distance model had the highest overall adjusted *R* ^2^ value (adj *R* ^2 ^= 0.205, *P *<* *0.001). However, the ratio of the spatial genetic variation explained by the resistance surface (Euclidean distance) and the variation explained by the sample locations (instead of patterns in the model) was lower than for the slope, peak NDVI, and RSF resistance surfaces, indicating a poorer model than these landscape variables (Galpern et al. 2014). Many of the habitat variables included in the RSF model were significant in univariate partial Mantel tests (peak NDVI, summer precipitation, slope; Table 2) and contributed to the top resistance model (elevation, open land cover, peak NDVI, annual mean precipitation, slope; Table 3); however, the RSF model itself was not significantly correlated with Dall's sheep gene flow (*r*_pm_ = −0.06, *P *=* *1.00) and had little support in the AICc model selection (*w*_i _= 0.00, Table 4). Some significant landscape variables included in the RSF model did not contribute to the top resistance model (e.g., rugged terrain), and summer precipitation was eliminated due to high VIF values (>15 in final candidate models), highlighting key differences underlying models predicting habitat selection and genetic connectivity.

Comparing the RSF and the top resistance models after partialling out the effects of geographic distance among individual sheep, the magnitude and direction were similar of the majority of the variables they had in common, with peak NDVI, slope, and open habitat types having a relatively high probability of selection and the strongest association with genetic distance. Areas of high annual precipitation were habitats Dall's sheep avoided and also had high resistance to gene flow. Mid‐range elevations (approximately 1200–2000 m) were selected habitats in the RSF, but had a negative correlation with gene flow.

## Discussion

Using RSFs allowed us to rank important habitat covariates which were constructive for building landscape genetics models. Additionally, RSF coefficients provided empirical and realistic values for resistance surface parameterization. These values are useful for comparison to hypothetical resistance surface values or those derived from expert opinion, and tested with genetic data to select the most parsimonious model. In this analysis, the best supported landscape resistance model for Dall's sheep gene flow differed from the resistance surface derived from summer habitat selection. While many of the RSF habitat variables were significant in predicting gene flow and contributed to the top model, the RSF model itself was not significantly correlated with Dall's sheep gene flow. Our results suggest that certain habitat features Dall's sheep selected during summer (rugged terrain, mid‐range elevation) did not influence effective dispersal or gene flow. Because the two methods assess different ecological functions at different temporal and spatial scales, using them in combination revealed key differences in home range and dispersal ecology of Dall's sheep which could be informative for guiding management actions.

### Ranking influences of genetic connectivity

Although both population‐ and individual‐based analyses identified the Wrangell and St. Elias ice fields and glaciers and the lower Chitina River valley as partial barriers to gene flow in sheep (Roffler et al. 2014), these large barriers were less correlated with genetic distance than topographic (steep slopes) and environmental features (open habitats, peak NDVI, and annual precipitation), illustrating the value of examining the influence of habitat components on population connectivity. The top resistance model included these landscape features and was a better predictor than barriers of gene flow in Dall's sheep.

An IBD genetic pattern was also previously detected in Dall's sheep (Roffler et al. 2014), attributed to the continuous nature of habitats in this region and relatively high population abundance (effective population size). Our landscape genetic results reveal that Dall's sheep in WRST can be characterized as having an IBD pattern; however, landscape resistance played a larger role than IBD in influencing genetic structure of the population. Dall's sheep populations in WRST exhibited weaker genetic structure (lower pairwise population genetic differentiation based on F_ST_ values) compared with populations with fragmented habitat and that have experienced declines (Luikart and Allendorf 1996; Valdez and Krausman 1999), suggesting that connectivity of preferred habitat, even outside of the breeding season, is relevant to maintaining genetic connectivity across large, montane landscapes. Thus, landscape characteristics in tandem with landscape configuration played a role in influencing Dall's sheep genetic connectivity.

Our optimization procedure was useful in parameterizing resistance surfaces, so we could rank landscape features in terms of influencing genetic connectivity. Accounting for uncertainty in the resistance values assigned to features theorized to affect gene flow is a major and evolving topic in landscape genetics (Spear et al. 2010). The optimization method has been demonstrated to be beneficial for identifying landscape features correlated with genetic connectivity, especially when also combined with a model selection approach (Wasserman et al. 2010; Tucker 2013). A potential drawback to optimization is a resulting model that is overfit, and therefore would perform poorly in predicting gene flow outside of the study area. We attempted to avoid this problem using a hypothesis‐driven framework for selection of landscape and environmental variables for which we hypothesized *a priori* to be of ecological importance to Dall's sheep across their range (Anderson and Burnham 2002; Richardson et al. 2016). This approach is considered beneficial because ensuing models will have better predictive power, which may be tested internally with a subset of the data, or externally with independent data (Hand et al. 2016; Richardson et al. 2016). Model testing presents a fruitful avenue for future landscape genetic analyses of this and other study systems.

### Influence of home range and breeding ecology on distribution

Similar to many vagile species, Dall's sheep commonly occupy different seasonal ranges, and selection of habitats during those time periods could reflect different ecological processes. Summer is the prime time of the year for northern ungulates to maximize their nutritional condition, and the late fall breeding season is when males and females interact, remaining highly sexually segregated for the remainder of the year (Geist 1971). It is, therefore, not surprising that different habitat features are important during different seasons. Rugged terrain was important in the summer habitat model, illustrating that alpine ungulates rely on escape terrain for predator avoidance, which is particularly important for females with offspring (Geist 1971). When groups intermingle during the prerut and rut seasons (October – early December), Dall's sheep distribution is likely influenced by availability of wind‐blown areas free of snow, due to ease of movement and access to forage.

Seasonal ranges of Dall's sheep are interrelated as the winter range is generally a contracted portion of the summer range (Hoefs and Cowan 1979), and thus, distribution during the breeding season is influenced by the general availability of habitat during the summer. Indeed, habitat selection models showed that Dall's sheep appear to be selecting areas within their annual range representing favorable conditions throughout the year (Appendix S1). Sheep have strong fidelity to seasonal ranges (Geist 1971), and although it is uncommon for sheep to make large movements or to permanently disperse, those that do may be more likely to travel across expanses of gentle topography between areas of rugged terrain that they inhabit for most of the year. A small number of such dispersers would be sufficient to account for levels of gene flow observed in this region (Roffler et al. 2014), and our resistance model for gene flow reflects this longer‐term process and habitat types that promote functional connectivity.

Differences in home range and breeding ecology highlight the influence of other factors on the distribution of wildlife species throughout the year. For example, resistance surfaces based on summer RSFs predicted genetic relatedness in Alaskan mountain goats (*Oreamnos americanus*) better than habitat models reflecting the period of the rut (Shafer et al. 2012). Differences were attributed to social behavior patterns of ungulates during breeding season, when habitat becomes less important to males than ensuring mating opportunities. Male mountain goats are known to make movements between female groups during the breeding season (Festa‐Bianchet and Côté 2008), and during this window of time, the habitat features important on a daily basis such as food and escape terrain may have a lower priority.

Indeed, high‐quality habitat for survival and reproduction does not necessarily translate to high‐quality dispersal habitat. Species may be capable of dispersal through low‐quality seasonal habitat with high resistance values based on indices of habitat suitability (Chetkiewicz and Boyce 2009; Creech et al. 2014). For example, a decrease in habitat suitability did not equate to higher landscape resistance measured by slower movement rates of cougars (*Puma concolor*; Dickson et al. 2005) or a decreased gene flow in capercaillie (*Tetrao urogallus;* Braunisch et al. 2010). Moreover, habitat use patterns within an organisms' home range will not necessarily reflect how landscape features influence dispersal and migration as demonstrated for bobcats (*Lynx rufus*; Reding et al. 2013), American marten (*Martes americana;* Wasserman et al.^2010^), and roe deer (*Capreolus capreolus*; Coulon et al. 2004). These studies underscore that movement behavior and resource use should not be confounded (Zeller et al. 2012)

### Management and conservation implications

Maintaining functional connectivity in species is vital for sustaining demographic and genetic processes and thus is important for long‐term population viability (Hanski and Gilpin 1997; Rudnick et al. 2012). More accurate parameterization of landscape genetics models will enable identification of important areas of habitat and population connectivity that are vital to maintaining gene flow, hence making it possible to target specific areas for conservation. It is especially critical to understand how connectivity could be impacted by landscape and climate change, and the implications for persistence of natural populations.

Determining whether habitat characteristics that contribute to higher resistance to movement and gene flow are primarily biotic or abiotic will have significance for designing models to predict the effects of climate change on the geographic range and connectivity of natural populations, and the responses of populations to environmental and landscape spatial heterogeneity over time (Rudnick et al. 2012). Here, we show that landscape components that are important for maintaining genetic connectivity among Dall's sheep are a combination of biotic (open habitats, peak NDVI) and abiotic (slope, elevation, precipitation) variables. Likewise, the best RSF model revealed biotic (open habitats, peak NDVI) and abiotic (slope, rugged terrain, elevation, precipitation) variables as the most explanatory for resource selection. Most abiotic variables are static in nature, and thus, the topographic component of sheep habitat will not be altered due to climate change. In particular, rugged terrain played a large role in predicting the probability of sheep habitat use and slope in predicting gene flow. Therefore, changes in climate patterns will likely have a relatively small effect on connectivity between populations of sheep based on these habitat types, which is favorable for long‐term persistence.

While it is not exactly clear how habitat distributions and configurations will shift over time as a result of changing climates, vegetation succession, and other ecological processes predictions based on climate models indicate a shift from open vegetation classes in alpine ecosystems to an upward expansion in shrub communities both in elevation and in latitude (Myers‐Smith et al. 2011). Thus, the biotic covariates included in the habitat and landscape genetics models such as open‐canopy vegetation may change over time. An increase in extent of woody shrubs could result in reduction and fragmentation of low‐elevation open habitat. Glaciers and persistent snow and ice at higher elevations are simultaneously receding, permitting an upward elevation shift in open vegetation, but will represent smaller habitat gains than is lost at low elevations. Hence, the overall amount of habitat loss or gain will depend on the rate of change of each habitat attribute. These predicted outcomes highlight that the effects of changing climate conditions on Dall's sheep and other northern ungulates will be complex, varying across spatial and temporal scales.

Alpine species may be particularly susceptible if changing climates result in a loss of functional connectivity and increasing isolation in high‐elevation habitats. Predicting the effects of landscape change on gene flow is an important planning tool, but modeling efforts must take into account different ecological functions reflected in seasonal habitat selection and long‐term effective dispersal. Consideration of landscape factors which influence both can help ensure that resources are managed to meet the survival needs of a species and to allow for long‐term connectivity, essential to effective conservation and management strategies for wildlife populations.

## Data accessibility

1. Survey locations: National Park Service Integrated Resource Management Applications (https://irma.nps.gov)
2. Microsatellite data: DRYAD http://dx.doi.org/10.5061/dryad.bq5rj Data files: dryad_ovda

## Supporting information


**Appendix S1.** Estimating the relative probability of Dall's sheep habitat use.
**Appendix S2.** Landscape coefficient resistance curves.
**Appendix S3.** Distance‐based Moran's eigenvector maps.
**Table S1.** Landscape and environmental variables used in Dall's sheep landscape genetics models, Wrangell‐St. Elias National Park and Preserve, Alaska.
**Table S2.** Parameter estimates presented as selection coefficients (β_1_) and standard errors (SE) for covariates in the top summer resource selection function model for Dall's sheep in Wrangell‐St. Elias National Park and Preserve, Alaska, 1983–2011.
