# Supplementary material for: Identification of landscape features influencing gene flow: How useful are habitat selection models?
Source: Evol Appl. 2016 Jun 3;9(6):805–17. doi: 10.1111/eva.12389 (PMC4908466; doi:10.1111/eva.12389)
Supplement: Supplementary file 1 — Appendix S1. Estimating the relative probability of Dall's sheep habitat use. Appendix S2. Landscape coefficient resistance curves. Appendix S3. Distance‐based Moran's eigenvector maps. Table S1. Landscape and environmental variables used in Dall's sheep landscape genetics models, Wrangell‐St. Elias National Park and Preserve, Alaska. Table S2. Parameter estimates presented as selection coefficients (β1) and standard errors (SE) for covariates in the top summer resource selection function model for Dall's sheep in Wrangell‐St. Elias National Park and Preserve, Alaska, 1983–2011. [file EVA-9-805-s001.docx]

**APPENDIX S1.** Estimating the relative probability of Dall’s sheep habitat use

*Survey data*

We used observations of sheep groups (*n* = 2,877) from aerial surveys conducted during 15 June – 8 August, 1983 – 2011 as the sample unit to characterize summer habitat selection. Observers classified and enumerated sheep into various sex and age categories including young, adult females (juvenile males included with females because they were not distinguishable from females by horn size and were generally located with female bands up to 2 years of age [Geist 1971, Strickland et al. 1992]), and adult males (>1/4 curl).

*Model development*

We estimated summer habitat selection of WRST sheep in the following categories: 1) all sheep, 2) males, and 3) females, with sub-categories of a) females with young, and b) females without young. We developed resource selection functions (RSFs; Boyce et al. 2002; Manly et al. 2002) using generalized logistic regression (GLM; Hosmer and Lemeshow 2000) to compare habitat at used and available sampling locations of each sheep group. We evaluated RSFs at the second-order scale (landscape; Johnson 1980), and our design corresponded to surveys with population level information about use and availability within seasonal home ranges.

We estimated the relative probability of use with the exponential approximation of the logistic regression model as:

*W* = exp (β_1_ *x*_1_ + β_2_ *x*_2_ +… + β_p_ *x*_p_) (eq.1)

where *W* is an index of the probability of use of a given site and β_1_ is the selection coefficient of resource variable *X*_1_ (Manly et al. 2002; Lele and Keim 2006; Johnson et al. 2006). We defined used habitat by the classified sheep group survey locations (*n* = 2,587), and available habitat as the area within a 7 km radius buffer established around all survey locations, including unclassified groups (*n* = 2,880; Fig.1). The 7 km buffer was chosen because it represents the radius of a 157 km^2^ circle, the area estimated to be the mean 95% adaptive kernel home range size for radiocollared male and female sheep in central Alaska (Burch and Lawler 2001). We randomly selected 13,200 points to represent available habitat.

We first used univariate logistic regression to assess nonlinear effects and to identify informative biotic and abiotic covariates to include in the multivariate models. We also used semi-parametric Generalized Additive Models (GAMs; Hastie and Tibshirani 1990) to evaluate distributions of covariates. Nonlinear covariates were incorporated as polynomials in the GLMs (Hosmer and Lemeshow 2000). We used a constrained model selection approach in order to consistently compare coefficients to test for differences in habitat use among sheep classes. Coefficients are not comparable unless the same set of parameters is used in a model because different combinations of covariates adjust their value (McCullagh and Nedler 1989; Hosmer and Lemeshow 2000).

We constructed a suite of competing *a-priori* multivariate logistic candidate models for each class to predict Dall’s sheep habitat selection. We used the conservative Bayesian information criterion (BIC; Schwarz 1978) to rank the models based on the BIC weights (*w_i_*) and calculate evidence ratios (the ratio between BIC weights) to assess the strength of evidence for a given model relative to a competing model (Anderson and Burnham 2002), with evidence ratio values >10 indicating strong support. We ranked the covariates based on the sum of their BIC weights (∑*w_i_*) from all models where the covariate was present following Burnham and Anderson (2002, pp. 167-168), and selected a consistent set of the highest ranked habitat covariates to build comparative RSF models. We randomly drew a balanced number of available points (same number as used points) for each sheep class and performed 100 iterations to estimate β_i_ values and standard errors.

*Spatial data*

Spatial and statistical analyses were conducted in the R statistical program (R Core Development Team 2013), and ArcMap 10.1 (ESRI 2012). We resampled GIS data with Spatial Analyst (ESRI 2012) to a 60 m^2^ cell resolution for analysis. We used a 60 m^2^ digital elevation model to derive elevation, slope, aspect and terrain ruggedness for each pixel across the study area. We used the Vector Ruggedness Measure (VRM) script for ArcGIS to calculate an index of ruggedness between 0 and 1 for each point based on vector dispersion of terrain aspect and angles in the 3 x 3 pixel neighborhood in comparison to the three-dimensional vector normal to each focal pixel (Sappington et al. 2007). Landcover classifications were based on attributes defined using multi-spectral image analysis of Landsat TM5 and TM7 data in combination with supporting field data, and made available at the 28.5 m^2^ pixel resolution by Stumpf (2008). Vegetation categorizations followed the Alaska Vegetation Classification system (Viereck et al. 1992). We used level II vegetation types and collapsed “mixed conifer-hardwood”, “conifer”, and “hardwood” into one category (conifer-hardwood). We characterize relative primary productivity of forage biomass with NDVI values (Pettorelli et al. 2005; Hebblewhite et al. 2008) obtained from 16-day composites at a 250m^2^ resolution from NASA’s Moderate Resolution Imaging Spectroradiometer (MODIS) satellites (Jenkerson et al. 2010). We used the mean maximum annual NDVI value for each location during 2001 – 2011 (which on average occurred 19-July). We obtained mean annual temperature and precipitation during 1971-2000 from the PRISM Climate Group (2009), and downscaled historical seasonal (June – August) estimates of temperature and precipitation decadal means were obtained from Scenarios Network for Alaska and Arctic Planning (SNAP; 2013) at the 771 m^2^ pixel spatial scale.

We screened for collinearity, excluding significantly correlated habitat covariates (*r* ≥ 0.7; Hosmer and Lemeshow 2000), or with variance inflation factors (VIF) > 10 (McCullagh and Nelder 1989), in which case the habitat covariate that explained a greater portion of the deviance was retained. Mean summer temperature and elevation were strongly correlated (*r* = -0.936), and mean summer temperature had a VIF value of 8.48, thus we removed it from further consideration. Comparisons of other habitat variables did not exceed the correlation threshold, and VIF values in multivariate models were within the acceptable tolerance range.

*Model validation*

We used a fivefold cross-validation procedure to assess the predictive capacity of used-available design RSF models, (Boyce et al. 2002). We reconstructed the top model for each sheep class using a random 80% subset of the data (training set), and used the remaining withheld 20% for evaluation (test set). We then compared the area-adjusted frequencies of test data predictions within 10 ranked bins to the observed bin rank for each RSF model. We assessed the model’s predictive performance with Spearman’s rank correlation coefficients (*r*_s_). A strong positive correlation, based on a higher proportion of used locations in the top ranked RSF bins, indicated higher predictive ability. To test for differences in resource selection among sheep classes, we first compared coefficients and 95% confidence intervals between the models. We then assessed the ability of each top model for a particular sheep class to predict resource selection of all other sheep classes by iteratively calculating *r*_s_ between the area-adjusted frequencies of each model training set and the test set.

Internal validation of resource selection models for all sheep classes using resampled training data performed well in the k-fold cross validations (*r*_s_ = 0.951 – 0.997, *P*< 0.001). These results indicate that despite some differences in selection coefficients among sheep classes, the combined RSF model may be used to predict the relative probability of resource selection in Dall’s sheep in WRST regardless of sex or reproductive status.

*Habitat selection*

Eight habitat covariates were consistently retained in best approximating models for all sheep classes (NDVI, open landcover [barren, sparse], elevation, ruggedness, slope, mean annual and summer precipitation) covariates, thus these 8 covariates made up the constrained model. The constrained model was the top ranking model for all sheep, all females, and females without young classes, the second ranked in the male class, and third for the females with young class.

Selection coefficients revealed that sheep selected for open habitat types and avoided closed landcover types (conifer hardwood forests, tall shrubs, and low shrubs). Ruggedness had the highest selection coefficient values (SI Table 2). Elevation and NDVI had a significant non-linear effect with Dall’s sheep largely selecting for mid-elevations (approximately 1,200 – 2,000 m) and intermediate NDVI values (0.2 – 0.6). The probability of selection decreased linearly among all sheep with increasing mean annual precipitation (range: β = -0.001 – -0.003, *P* < 0.001), but had a non-linear relationship with summer precipitation, as sheep disproportionally selected areas between approximately 20 and 40 cm of precipitation a year.

**APPENDIX S2**. Landscape coefficient resistance curves

We estimated RSF selection coefficients (βi) using equation 1, and used resulting coefficient values (SI Table 2), scaled from 1 to the maximum resistance value (Rmax = 2, 5, 10, 25, 50, 100, 200, 500), and calculated the inverse of the habitat selection component to represent resistance using ArcGIS Spatial Analyst Raster Calculator in ArcMap 10.1 (ESRI 2012).

R = (R_max_+1*((e ^β^_i_ - 1) /(R_max_ - 1)))^-1^

**APPENDIX S3**. Distance-based Moran’s eigenvector maps

We used distance-based Moran’s eigenvector maps (MEM; Dray et al. 2012; Legendre and Legendre, 2012) to identify the proportion of spatial genetic variation explained by each of the landscape resistance surfaces assessed in the multiple regression of distance matrices (MRDM) models in addition to the Euclidean distance model. The MEM eigenvectors were calculated using principal coordinate analysis (PCoA) of a truncated geographic distance matrix among the Dall’s sheep genetic sampling locations (Dray et al. 2006) using the MEMGENE R package (Galpern et. al 2014). The MEM eigenvectors are a set of orthogonal variables describing possible patterns spatial autocorrelation in the genetic data at different scales. Genetic patterns were modeled using a genetic distance matrix with the proportion of shared alleles metric (D_ps_; Bowcock et al. 1994) as the dependent variable and the MEM eigenvectors as predictor variables. All positive MEM eigenvectors were entered into a model and a forward selection procedure was performed with permutation tests to identify variables describing significant spatial patterns. Forward selection was stopped when the addition of a variable did not improve the model fit, measured by a significant (α = 0.05) increase of the adjusted *R^2^* at each step. The proportion of variance in genetic data explained by eigenvectors was assessed using PCoA, and the variables (individual scores on the PCoA axes) were ordered in terms of the amount of variation they explained.

**SI Table 1.** Landscape and environmental variables used in Dall’s sheep landscape genetics models, Wrangell-St. Elias National Park and Preserve, Alaska.

| Feature | Description | Hypothesized resistance | Ecological rationale | Optimized resistance |
| --- | --- | --- | --- | --- |
| RSF | Continuous variable: inverse values of summer resource selection function model | Habitats with a high probability of selection will be more conducive for gene flow than habitat types Dall's sheep avoid | Habitat models can be used to predict levels of relatedness (Shaffer et al. 2012) and gene flow (Weckworth et al. 2013) | 25 |
| Elevation | Continuous variable: range in study area 40-5,000 m. | Resistance has a curvilinear shape increasing with high and low elevations | Dall's sheep prefer mid-elevations. Geist 1971, Hoefs 1984, Nichols and Bunnell 1999, Rachlow and Bowyer 1998, Appendix S1 | 100 |
| Land cover | Categorical variable: open (barren, dwarf shrub, sparse, herbaceous) and closed (low and tall shrub, conifer-hardwood forest) | Forested land cover has high resistance to gene flow, open types have no resistance | Thinhorn sheep avoid forests/dense shrubs and select open habitat types. Walker et al. 2007, Appendix S1 | 25 |
| Peak NDVI | Continuous variable: 0 – 1, mean maximum annual NDVI value 2001 – 2011 (mean date July 19) | Resistance will be highest in high and low NDVI areas, and lower with moderate NDVI | Sheep distribution is highly influenced by the availability of adequate forage. Bunnell 1978, Hoefs and Cowan 1979, Hoefs 1984, Hoefs and Nowlan 1997, Nichols and Bunnel 1999. Sheep distribution is correlated with mid-range values of NDVI. Appendix S1, Terwilliger 2005. | 50 |
| Precipitation (annual) | Continuous variable: mean annual precipitation 1971-2000 | Resistance will be highest in areas of high annual precipitation; majority deposited as winter snow. | Areas of heavy precipitation in the winter results in deep snow, limiting movements and access to forage in the winter. Burles and Hoefs 1984, Nichols 1978, Rachlow and Bowyer 1998 | 5 |
| Precipitation (summer) | Continuous variable: mean precipitation June - August, 1971-2000 | Areas of very low and high precipitation will have high resistance | Summer precipitation positively influences sheep habitat use by increasing primary productivity of summer range. Bunnell 1978, Hik and Carey 2000, Hoefs and Cowan 1979 | 50 |
| Rugged terrain | Continuous variable:Vector Ruggedness Measure | Resistance has a curvilinear shape increasing with high and low ruggedness index values | Thinhorn sheep use moderately rugged areas as escape terrain (provides safety from predators). Geist 1971, Rachlow and Bowyer 1998, Walker et al. 2007, Appendix S1 | 100 |

| **SI Table 1.** Continued | | | | |
| --- | --- | --- | --- | --- |
| Slope | Continuous variable | Resistance has a curvilinear shape increasing with high and low ruggedness index values | Sheep favor steep slopes. Geist 1971, Hoefs 1984, Nichols and Bunnell 1999, Rachlow and Bowyer 1998, Walker et al. 2007 | 50 |
| Snow cover  (Oct 1) | Categorical variable: mean continual snow cover beginning Oct. 1 (2001-2012) | Snow-free areas have no resistance; snow cover has high resistance | >30 cm impedes sheep movement. Dall’s sheep begin moving to their breeding grounds in late September prior to the peak of the rut in late November. Burles and Hoefs 1984, Hoefs and Cowan 1979, Hoefs 1984, Barichello and Carey 1988, Rachlow and Bowyer 1998, Summerfield 1974. | 100 |
| Glaciers | Categorical variable: all ice features | Ice features have high resistance to sheep movement | Landscape feature could be barrier to gene flow. Roffler et al. 2014 | 100 |
| Chitina River | Categorical variable: ≤ 1,000 m elevation and > 10 km wide | River valley has high resistance | Thinhorn sheep generally avoid low elevation, forested river valleys between mountain ranges. Nichols and Bunnell 1999, Walker et al. 2007 | 100 |
| Lower Chitina River | Categorical variable: (≤ 1,000 m elevation and > 20 km wide) | River valley has high resistance | Landscape feature could be barrier to gene flow. Roffler et al. 2014 | 50 |

**SI Table 2**. Parameter estimates presented as selection coefficients (β_1_) and standard errors (SE) for covariates in the top summer resource selection function model for Dall’s sheep in Wrangell-St. Elias National Park and Preserve, Alaska, 1983-2011.

|  | β_1_ | SE |  | *P* |  |
| --- | --- | --- | --- | --- | --- |
| Elevation | 0.001 | <0.001 |  | 0.00  0 |  |
| Elevation ^2^ | 0.000 | <0.001 |  | 0.00 |  |
| Annual precipitation | -0.002 | <0.001 |  | 0.00 |  |
| Slope | 0.058 | <0.001 |  | 0.00 |  |
| Slope ^2^ | 0.000 | <0.001 |  | 0.00 |  |
| Rugged terrain | 7.108 | 0.018 |  | 0.00 |  |
| Rugged terrain ^2^ | -18.090 | 0.050 |  | 0.00 |  |
| Barren (open vegetation) | 0.034 | 0.002 |  | 0.04 |  |
| Sparse (open vegetation) | 0.302 | 0.002 |  | 0.00 |  |
| Summer precipitation | 0.033 | <0.001 |  | 0.00 |  |
| Summer precipitation | 0.000 | <0.001 |  | 0.00 |  |
| Peak NDVI | 8.326 | 0.008 |  | 0.00 |  |
| Peak NDVI^2^ | -7.890 | 0.010 |  | 0.00 |  |

**SI Literature Cited**

Anderson, D. R. and K. P. Burnham. 2002. Avoiding Pitfalls When Using Information-Theoretic Methods. The Journal of Wildlife Management **66**:912–918.

Barichello, and N., J. Carey. 1988. Snow depth as a likely factor contributing to the decline of a sheep population in the central Yukon. Biennial Symposium Northern Wild Sheep and Goat Council **6**:282.

Bowcock, A., A. Ruiz-Linares, J. Tomfohrde, E. Minch, J. Kidd, and L. Cavalli-Sforza. 1994. High resolution of human evolutionary trees with polymorphic microsatellites. Nature **368**:455-457.

Boyce, M.S., P.R. Vernier, S.E. Nielsen, and F.K. Schmiegelow 2002. Evaluating resource selection functions. Ecological Modelling **157**:281–300.

Bunnell, F.L. 1978. Horn Growth and Population Quality in Dall Sheep. The Journal of Wildlife Management **42**:764–775.

Burch, J. and J. Lawler. 2001. Ecology and demography of Dall’s sheep in Yukon-Charley Rivers National Preserve: identifying critical Dall’s sheep habitat and habitat use patterns. Yukon-Charley Rivers National Preserve, Alaska. Technical Report NPS/AR/NRTR-2001/39. United States Department of the Interior, National Park Service, Alaska Region.

Burles, D.W. and M. Hoefs. 1984. Winter mortality of Dall sheep, *Ovis dalli dalli*, in Kluane National Park, Yukon. Canadian Field-Naturalist **98**:479–484.

Dray, S., P. Legendre, and P.R. Peres-Neto. 2006. Spatial modelling: a comprehensive framework for principal coordinate analysis of neighbour matrices (PCNM). Ecological Modelling **196**:483–493.

Dray, S., R. Pélissier, P. Couteron, M.J. Fortin, P. Legendre, P.R. Peres-Neto, E. Bellier, E., et al. 2012. Community ecology in the age of multivariate multiscale spatial analysis. Ecological Monographs **82**:257–275.

Galpern, P., P.R. Peres-Neto, J. Polfus, and M. Manseau. 2014. MEMGENE: Spatial pattern detection in genetic distance data. Methods in Ecology and Evolution **5**: 1116–1120.

Geist, V. (1971) Mountain sheep. University of Chicago Press, Chicago.

Hastie, T. and R.J. Tibshirani. 1990. Generalized additive models. Chapman & Hall. London Hosmer, D.W., S. Lemeshow. 2000. Applied Logistic Regression, 2nd edn. Wiley, New York

Hebblewhite, M., E. Merrel and G. McDermid. 2008. A multi-scale test of the forage maturation hypothesis in a partially migratory ungulate population. Ecological Monographs **78**:141–166.

Hik, D.S. and J. Carey. 2000. Cohort variation in horn growth of Dall sheep rams in the southwest Yukon, 1969-1999. Biennial Symposium Northern Wild Sheep and Goat Council **12**:88–100.

Hoefs, M. and I.M. Cowan. 1979. Ecological investigations of a population of Dall sheep (*Ovis dalli dalli* Nelson). Syesis **12**:1–81.

Hoefs, M. 1984. Productivity and Carrying Capacity of a Subarctic Sheep Winter Range. Arctic **37**:141–147.

Hoefs, M. and U. Nowlan. 1997. Comparison of horn growth in captive and free-ranging Dall’s rams. Journal of Wildlife Management **61**:1154–1160.

Hosmer, D.W. and S. Lemeshow. 2000. Applied Logistic Regression, 2nd edn., Wiley, New York.

Jenkerson, C.B., T. Maiersperger, and G. Schmidt. 2010. eMODIS: A user-friendly data source: U.S. Geological Survey Open-File Report 2010–1055. U.S. Geological Survey, EROS Center, Sioux Falls, SD. http:pubs.usgs.gov/of/2010/1055/. Accessed 20 June 2013.

Johnson, D.H. 1980. The comparison of usage and availability measurements for evaluating resource preference. Ecology **61**:65–71.

Johnson, C. J., S. E. Nielsen, E. H. Merril, T. L. McDonald and M. S. Boyce. 2006. Resource Selection Functions Based on Use – Availability Data : Theoretical Motivation and Evaluation Methods. Journal of Wildlife Management **70**:347–357.

Legendre, P. and L. Legendre, 2012. Numerical Ecology, 3rd. edn. Elsevier Limited, Oxford.

Lele, S.R. and J.L. Keim. 2006. Weighted distributions and estimation of resource selection probability functions. Ecology **87**:3021–3028.

Manly, B.F.J., L.L. McDonald, D.L. Thomas, T.L. McDonald, and W.P. Erickson. 2002. Resource Selection by Animals Statistical Design and Analysis for Field Second Edition.

McCullough, P. and J.A. Nelder. 1989. Generalized linear models. 2nd edn. Chapman and Hall, London, UK.

Nichols, L. 1978. Dall Sheep Reproduction. Journal of Wildlife Management **42**:570–580.

Nichols, L., and F. L. Bunnell. 1999. Natural history of thinhorn sheep. Mountain sheep of North America. (eds R. Valdez and P.R. Krausman), pp. 23-77. University of Arizona Press, Tucson, Arizona.

Pettorelli, N., J. O. Vik, A. Mysterud, J.-M. Gaillard, C. J. Tucker and N. C. Stenseth. 2005. Using the satellite-derived NDVI to assess ecological responses to environmental change. Trends in Ecology and Evolution **20**:503–10.

PRISM Climate Group, Oregon State University and Alaska Region Inventory and Monitoring Program, National Park Service; accessed 1 Dec. 2009 science.nature.nps.gov/nrdata

Rachlow, J.L. and R.T. Bowyer. 1998. Habitat selection by Dall’s sheep (*Ovis dalli*): maternal trade-offs. Journal of Zoology **245**:457–465.

Roffler, G.H., S.L. Talbot, G. Luikart, G.K. Sage, K. Pilgrim, L.G. Adams, and M.K. Schwartz 2014. Lack of sex-biased dispersal promotes fine-scale genetic structure in alpine ungulates. Conservation Genetics **15**:837-851.

Sappington, J. M., K. M. Longshore and D. B. Thompson. 2007. Quantifying Landscape Ruggedness for Animal Habitat Analysis: A Case Study Using Bighorn Sheep in the Mojave Desert. Journal of Wildlife Management **71**:1419–1426.

Shafer, A.B.A., J.M. Northrup, K.S. White, M.S. Boyce, S.D. Côté, and D.W. Coltman. 2012. Habitat selection predicts genetic relatedness in an alpine ungulate. Ecology **93**:1317–29.

SNAP, University of Alaska. (2013) Retrieved June 1 2013 from <http://www.snap.uaf.edu/data.php>

Strickland, D., L. McDonald, K. Taylor, K. Jenkins and J. Kern. 1992. Estimation of Dall sheep numbers in the Wrangell-St. Elias National Park and Preserve. Pp. 237–255 in Biennial Symposium of the Northern Wild Sheep and Goat Council.

Stumpf, K. 2008. Wrangell-St. Elias National Park and Preserve Landcover Mapping Project. National Park Service, Natural Resource Technical Report NPS/WRST/NRTR—2008/095. D-101.

Terwilliger, M.L.N. 2005. Population and habitat analyses for Dall’s sheep (*Ovis dalli dalli*) in Wrangell-St. Elias national Park and Preserve. M.S. Thesis. University of Alaska. Fairbanks, Alaska.

Viereck, L. A., C. T. Dyrness and A. R. Batten. 1992. The Alaska Vegetation Classification.

Walker, A.B.D., K.L. Parker, M.P. Gillingham, D.D. Gustine, and R.J. Lay. 2007. Habitat selection by female Stone’s sheep in relation to vegetation, topography, and risk of predation. Ecoscience **14**:55–70.

Weckworth, B. V, M. Musiani, N.J. Decesare, A.D. McDevitt, M. Hebblewhite, and S. Mariani. 2013. Preferred habitat and effective population size drive landscape genetic patterns in an endangered species. Proceedings of the Royal Society of Biological Sciences **280**:20131756.
